# A Supramodal Neural Network for Speech and Gesture Semantics: An fMRI Study

**DOI:** 10.1371/journal.pone.0051207

**Published:** 2012-11-30

**Authors:** Benjamin Straube, Antonia Green, Susanne Weis, Tilo Kircher

**Affiliations:** 1 Department of Psychiatry and Psychotherapy, Philipps-University Marburg, Marburg, Germany; 2 Department of Psychiatry and Psychotherapy, RWTH Aachen University, Aachen, Germany; 3 Department of Neurology, RWTH Aachen University, Aachen, Germany; 4 Department of Psychology, Durham University, Durham, United Kingdom; University Of Cambridge, United Kingdom

## Abstract

In a natural setting, speech is often accompanied by gestures. As language, speech-accompanying iconic gestures to some extent convey semantic information. However, if comprehension of the information contained in both the auditory and visual modality depends on same or different brain-networks is quite unknown. In this fMRI study, we aimed at identifying the cortical areas engaged in supramodal processing of semantic information. BOLD changes were recorded in 18 healthy right-handed male subjects watching video clips showing an actor who either performed speech (S, acoustic) or gestures (G, visual) in more (+) or less (−) meaningful varieties. In the experimental conditions familiar speech or isolated iconic gestures were presented; during the visual control condition the volunteers watched meaningless gestures (G−), while during the acoustic control condition a foreign language was presented (S−). The conjunction of the visual and acoustic semantic processing revealed activations extending from the left inferior frontal gyrus to the precentral gyrus, and included bilateral posterior temporal regions. We conclude that proclaiming this frontotemporal network the brain's core language system is to take too narrow a view. Our results rather indicate that these regions constitute a supramodal semantic processing network.

## Introduction

Comprehension of natural language is a complex capacity, depending on several cognitive and neural systems. Over the last years knowledge of the brain processes underlying single word and sentence processing has grown by examining phonological, semantic and syntactic/sentence processing networks. But not only speech is a communicative source, features such as tone of voice, facial expression, body posture, and gestures also transmit meaning that has to be decoded. Whether such meaning derived from speech and gesture is (at least partly) represented in a common neural network is an important question to better understand the neural organization of semantics and especially its flexible utilization for communication. Therefore, this study investigates whether there is a brain network common to the processing of both speech and gesture semantics.

There is consensus that brain regions crucial for the processing of spoken or written language are the left inferior frontal gyrus (LIFG), the left temporal cortex, and their homologues in the right hemisphere [1]–[3]. Retrieval of semantic information, the processing of semantic relations between words and the processing of syntax in sentences have been related to the LIFG (especially BA 44/45 and 47) [1], [4], [5]. The left temporal cortex is stronger involved in sentential semantic processing than in syntactic processing. Especially posterior aspects of the middle temporal gyrus (MTG) and the inferior temporal gyrus (ITG) have been linked to the interpretation of meaning on a sentence level [6], detection of semantic anomalies [7], and maintenance of conceptual information [8], [9], with also the right hemispheric homologue areas being involved [10]. These findings are independent of the input modality, i.e. whether the language is presented auditorily (spoken) or visually (written) [11], [12].

From behavioral studies it is known that gestures indeed do convey meaning. Several studies using event-related potentials were able to show that gestures induce electrophysiological correlates of semantic processing [13]–[17]. Except pantomimes (i.e. acting out a whole sequence of information) and emblems (highly conventionalized symbols as the thumbs up-gesture), all kinds of gestures are produced together with speech. However, without accompanying speech the meaning of most gestures is not fixed [18], [19]. Concerning the neural correlates of gesture processing without sentence context, several studies have contrasted the viewing of meaningful complex gestures, such as emblems, to that of meaningless gestures. Interestingly, the regions commonly observed are the LIFG including Broca's area (BA 44, 45, 47), as well as the left middle temporal gyrus (MTG; BA 21; [20]–[22]). This activity was interpreted as the mapping of symbolic gestures and spoken words onto common, corresponding conceptual representations.

Further support for the idea that gesture semantics might be processed in the same network as spoken language comes from studies on sign language processing. Sign languages (SL) can convey the same information contained in speech, but have visuospatial properties similar to the properties of coverbal gestures. Comparable to the results from spoken language processing, neuroimaging studies on SL comprehension indicate a crucial role for the left superior temporal gyrus/sulcus and the LIFG (e.g., [23], [24]).

Lastly, there is a growing number of studies examining the processing of gestures in context of speech, highlighting the importance of inferior frontal, posterior temporal and inferior parietal regions (e.g., [25]–[33]). Based upon the studies available it seems justified to conclude that semantic processing of gestures and semantic processing of speech activates an overlapping neural network involving inferior frontal and posterior temporal regions.

The neural basis of gesture-speech interactions is investigated by an increasing number of functional magnetic resonance imaging (fMRI) studies [25]–[39]. These studies predominantly focussed on the processing of iconic coverbal gestures, suggesting that the left posterior temporal cortex is especially relevant for the integration of iconic gestures and the corresponding sentence context [25], [28], [31], [32]. However, left inferior frontal and parietal brain activations were reported for mismatches between unrelated concrete speech and iconic gesture information [25], [29]. Although these studies focussed on the interaction of speech and iconic gesture semantics, common activation patterns for the processing of iconic gestures and speech semantics have not specifically been investigated. In contrast to emblems and pantomimes, iconic gestures are less conventionalized and usually accompany speech. While emblems are socially transmitted and function like learned vocabulary, iconic gestures are shaped individually by speaker's needs. They share a formal relationship with the co-occurring speech content in that they illustrate forms, shapes, events or actions that are the topic of the simultaneously occurring speech. Since without accompanying speech the meaning of iconic gestures is not fixed [18], [19] it is unknown if a supramodal network, as demonstrated for symbolic gestures and speech [21], also exists for the comprehension of less language-like stimuli like iconic gestures.

According to the “Feature Integration Model (FIM)” for gesture-speech comprehension proposed by Obermeier, three levels of processing can be divided: 1) The perceptual analysis, 2) feature extraction and 3) integration and higher order cognitive influences ([35]; page 136). Within this model it has been assumed, that the processing of gesture and speech interacts on all processing levels. On the feature extraction level, visual features (e.g., hand shape, trajectory and its meaning) are extracted from gestures and auditory features are extracted from speech (e.g., word form, word category and lemma [semantic meaning]). Yet on this feature extraction level the model predicts interactions between modalities. Thus one could fancy that gesture information facilitates decisions about word category [35]. Assuming that these interactions are based on – at least partly – overlapping activated semantic nodes of a supramodal semantic network, the model predicts common neural correlates of speech and gesture semantics. However, up to now little is known about audio-visual communalities or interactions for iconic gestures and corresponding speech on this intermediate processing level [35].

Based on the findings for language, symbolic gesture and co-verbal gesture processing we suppose that the processing of semantics decoded from speech and iconic gesture input, respectively, depends on a common network of left-lateralized inferior frontal regions (especially BA 45, 47) and posterior temporal regions (MTG, ITG). To test this hypothesis, we conducted a functional imaging study that investigated the neural convergence sites of the processing of spoken semantics and iconic gesture semantics in the human brain. We used multiple baseline conditions to optimize interpretation of the functional imaging data [40], i.e. we contrasted familiar speech (German) to an unknown language (Russian) and compared meaningful iconic gestures depicting shapes or movements to equally complex but very diffuse arm and hand movements.

## Methods

### Ethics Statement

All subjects gave written informed consent prior to participation in the study. The study was approved by the local ethics committee.

### Participants

Eighteen healthy male subjects participated in the study. Due to excessive head movement two subjects had to be excluded. The mean age of the remaining 16 subjects was 28.8 years (SD: 8.3, range 23.0–55.0). All participants were right handed [41], native German speakers and had no knowledge of Russian. All subjects had normal or corrected-to-normal vision, none reported any hearing deficits. Exclusion criteria were a history of relevant medical or psychiatric illness of the participants or in his first degree relatives.

### Stimulus Material

**Figure 1:**
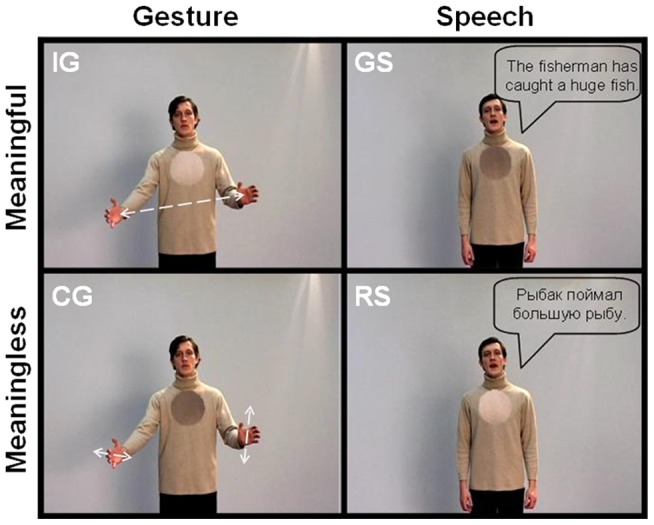
Design with examples of the meaningful (iconic gesture: G+; german sentence: S+) and meaningless (control gesture: G−; russian sentence: S−) speech and gesture video stimuli. The stimulus material consisted of video clips of an actor either speaking or performing gestures (exemplary screenshots). Speech bubbles (translations of the original German sentence “Der Fischer hat einen großen Fisch gefangen”) are inserted for illustrative purposes only. Note the dark- and light-colored spots on the actor's sweater that were used for the control task. The actor displayed in the photograph has given written informed consent to the publication of his photograph.

The details of the stimulus production are further described in Green et al. (2009). For the current analysis a set of (32 per condition ×4 conditions (out of 8, see [25]×4 sets) short video clips depicting an actor was used: 1) German sentences without gestures [S+], 2) Russian sentences without gestures [S−], 3) iconic gestures without speech [G+], and 4) less meaningful control gestures without speech [G−] (Figure 1). Thus, we presented videos with isolated speech or isolated gesture elements, both of them in either a high meaning or a low meaning variety.

We decided to contrast familiar speech (German) to speech in an unknown language (Russian) as a high level baseline contrast. By doing so we were able to subtract out all those activations related to sublexical processing, but nevertheless presented natural speech. All sentences had a similar grammatical structure (subject – predicate – object) and were translated into Russian. Words that sounded similar in each language were avoided. Examples for the German sentences are: ‘The fisherman has caught a **huge** fish’ (“Der Angler hat einen großen Fisch gefangen”), ‘The cottage is on a very **high** mountain’ (“Die Hütte ist auf einem sehr hohen Berg”) or ‘The table in the kitchen is **round**’ (“Der Tisch in der Küche ist rund”). Thus, the sentences had a similar length of five to eight words and a similar grammatical form, but differed considerable in content. The corresponding gestures (keyword indicated in bold) had to match McNeill's definition of ‘iconic gestures’ in that they illustrated the form, size or movement of something concrete that is usually mentioned in speech [42]. For each meaningful gesture we developed a diffuse gesture, which was comparable in complexity and movement characteristics but contained no semantic information.

The same male bilingual actor (German and Russian) performed all the utterances and gestures in a natural spontaneous way. Intonation, prosody and movement characteristics in the corresponding variations of one item were closely matched. At the beginning and the end of each clip the actor stood with arms hanging comfortably. Each clip had a duration of 5 s including 500 ms before and after the experimental manipulation, where the actor neither speaks nor moves. In the present study the semantic aspects of the stimulus material refer to differences between iconic versus meaningless gestures (without speech) and German versus Russian sentences (without gestures).

For stimulus validation, 20 participants not taking part in the fMRI study rated each video on a scale from 1 to 7 on understandability, imageability and naturalness (1 =  very low to 7 =  very high). In order to assess understandability participants were asked: How understandable is the video clip? (original: “Wie VERSTÄNDLICH ist dieser Videoclip?”). The rating scale ranged from 1 =  very difficult to understand (sehr schlecht verständlich) to 7 =  very easy/good to understand (sehr gut verständlich). For naturalness ratings the participants were asked: How natural is the scene? (original: “Wie NATÜRLICH ist diese Szene?”). The rating scale ranged from 1 =  very unnatural (sehr unnatürlich) to 7 =  very natural (sehr natürlich). Finally, for judgements of imageability the participants were asked: How pictorial/imageable is the scene? (original: “Wie BILDHAFT ist dieser Videoclip?”). The rating scale ranged from 1 =  very abstract (sehr abstrakt) to 7 =  very pictoral/imageable (sehr bildhaft). These scales have been used in previous investigations, too [25]–[28], [39], [43], [44]. Other parameters such as movement characteristics, pantomimic content, transitivity or handedness were coded by two of the authors (B.S., A.G.). A set of 1024 video clips (128 German sentences with iconic gestures and their counterparts in the other seven conditions) were chosen as stimuli for the fMRI experiment on the basis of high naturalness and comparability of movement characteristics (across conditions), as well as high understandability for the German conditions. The stimuli were divided in four sets in order to present each participant with 256 clips during the scanning procedure (32 items per condition), counterbalanced across subjects. Across subjects each item was presented in all four conditions but a single participant only saw complementary derivatives of one item, i.e. the same sentence or gesture information was only presented once per participant. This was done to prevent from speech or gesture repetition or carryover effects. Again, all parameters listed above were used for an equal assignment of the video clips to the four experimental sets, to avoid set-related between-subject differences.

The ratings on understandability for the four conditions used in this study clearly show the intended main effect of meaning, with the meaningful varieties scoring higher than the control varieties (F(1,508)  = 3925.93, P<0.001, partial-eta-squared  = 0.885; two-factorial within-subjects ANOVA). Video clips with German speech scored higher than 6 and Russian sentences scored lower than 3 on understandability (S+ m = 6.59, SD  = 0.18; S− m = 1.19, SD  = 0.22; S+>S−: T(254)  = 214.104, P<0.001). Concerning the gestures this difference was less strongly pronounced but still present (G+ m = 3.12, SD  = 0.89; G− m = 2.25, SD  = 0.64; G+>G−: T(254)  = 8.972, P<0.001). In addition to the main effect of meaning, we also revealed a main effect of modality (Speech > Gesture; F(1,508)  = 580.17, P<0.001, partial-eta-squared  = 0.533) as well as an interaction (F(1,508)  = 2038.44, P<0.001, partial-eta-squared  = 0.801), indicating that the difference between S+ and S− is more pronounced than the difference G+ vs. G−. These results are in line with the assumption that when presented without the respective sentence context isolated iconic gestures are less meaningful, but even then they still transport more meaning than the control gestures, indicating that our manipulation was effective.

The meaningful varieties scored higher than the control varieties in the rating of naturalness (F(1,508)  = 467.02, P<0.001; main effect; partial-eta squared  = 0.479). Post-hoc tests indicated that the meaningful varieties were perceived as equally natural, whereas all other comparisons revealed significant differences (all P<0.001; S+ m = 3.61, SD  = 0.33; S− m = 2.67, SD  = 0.21; G+ m = 3.59, SD  = 0.60; G− m = 2.88, SD  = 0.47). In addition to the main effect of naturalness, we also revealed a main effect of modality (F(1,508)  = 6.172, P<0.013; partial-eta-squared  = 0.012), indicating gesture stimuli scored higher than speech stimuli in the rating of naturalness. Finally we obtained interaction between modality and meaning (F(1,508)  = 8.98, P<0.003; partial-eta squared  = 0.017). The rather low naturalness ratings may be explained by the fact that isolated speech or gesture segments are relatively uncommon in daily life.

Imageability ratings indicated that there were also differences between the conditions concerning their property to evoke mental images. Again the meaningful varieties scored higher than the control varieties (F(1,508)  = 2081.46, P<0.001; all post-hoc tests significant at P<0.001; partial-eta squared  = 0.804). Highest imageability was assigned to German speech and the lowest to Russian speech (S+ m = 4.33, SD  = 0.30; S− m = 1.17, SD  = 0.14; G+ m = 3.78, SD  = 0.77; G− m = 2.89, SD  = 0.56). Thus, we obtained also a main effect modality (F(1,508)  = 173.131, P<0.001; main effect; partial-eta-squared  = 0.254), indicating gesture stimuli scored higher than speech stimuli in the rating of imagebility, and an interaction between modality and meaning (F(1,508)  = 653.833, P<0.001; partial-eta squared  = 0.563).

The sentences had an average speech duration of 2269 ms (SD  = 383 ms), with German sentences being somewhat longer than Russian sentences (S+ m = 2330 ms, SD  = 343 ms; S− m = 2208 ms, SD  = 413 ms; F(1,254)  = 6.619, P<0.05; partial-eta squared  = 0.025). The gestures analyzed here had an average gesture duration of 2770 ms (SD  = 462 ms) and did not differ between meaningful and diffuse gestures (G+ m = 2755 ms, SD  = 475 ms; G− m = 2784 ms, SD  = 449 ms; F(1,254)  = 0.237, P = 0.627).

Events for the fMRI statistical analysis were defined in accordance with the bimodal conditions (reported in [25]) as the moment with the highest semantic correspondence between speech and the gesture stroke (peak movement): Each sentence contained only one element that could be illustrated, which was intuitively done by the actor. The events occurred on average 2142 ms (SD  = 538 ms) after the video start and were used for the modulation of events in the event-elated fMRI analysis. The use of these predefined integration time points (reported in [25]) for the fMRI data analysis has the advantage that the timing for all conditions of one stimulus is identical since conditions were counterbalanced across subjects.

### fMRI data acquisition

All MRI data were acquired on a Philips Achieva 3T scanner. Functional images were acquired using a T2-weighted echo planar image sequence (TR  = 2 seconds, TE  = 30 ms, flip angle 90°, slice thickness 3.5 mm with a 0.3-mm interslice gap, 64×64 matrix, FoV 240 mm, in- plane resolution 3.5×3.5 mm, 31 axial slices orientated parallel to the AC-PC line covering the whole brain). Four runs of 330 volumes were acquired during the experiment. The onset of each trial was synchronized to a scanner pulse.

### Experimental design and procedure

An experimental session comprised 256 trials (32 for each condition) and consisted of four 11-minute blocks. Each block contained 64 trials with a matched number of items from each condition. The stimuli were presented in an event-related design in pseudo-randomized order and counterbalanced across subjects. As described above (stimulus material) across subjects, each item was presented in all conditions but a single participant only saw complementary derivatives of one item, i.e. the same sentence or gesture information was only seen once per participant. This was done to prevent speech or gesture repetition or carry over effects. Each clip was followed by a fixation cross on grey background with a variable duration of 3750 ms to 6750 ms (average: 5000 ms).

Before scanning, each participant received at least 10 practice trials outside the scanner, which were different from those used in the main experiment. Before the experiment started, the volume of the videos was individually adjusted so that the clips were clearly audible. During scanning, participants were instructed to watch the videos and to indicate via left hand key presses at the beginning of each video whether the spot displayed on the actor's sweater was light or dark. This task enabled us to investigate implicit speech and gesture processing without possible instruction-related attention biases. Performance rates and reaction times were recorded.

### MRI data analysis

MR images were analyzed using Statistical Parametric Mapping standard routines and templates (www.fil.ion.ucl.ac.uk). After discarding the first five volumes to minimize T1-saturation effects, all images were spatially and temporally realigned, normalized (resulting voxel size 4×4×4 mm^3^), smoothed (10 mm isotropic Gaussian filter) and high-pass filtered (cut-off period 128 s).

Statistical whole-brain analysis was performed in a two-level, mixed-effects procedure. In the first level, single-subject BOLD responses were modeled by a design matrix comprising the onsets of each event (see stimulus material) of all eight experimental conditions. The hemodynamic response was modeled by the canonical hemodynamic response function (HRF) and its temporal derivative. The volume of interest was restricted to grey matter voxels by use of an inclusive mask created from the segmentation of the standard brain template. Parameter estimate (ß−) images for the HRF were calculated for each condition and each subject. As SPM5 provides optimized second level models we used SPM5 for a random-effects group analysis. Parameter estimates for the four relevant conditions were entered into a within-subject one-way flexible factorial ANOVA. The semantic aspects of language processing were isolated computing the difference contrast of German versus Russian sentences [S+>S−], whereas the semantic aspects of action processing were revealed by contrasting meaningful gestures against control gestures [G+>G−]. Both these contrasts were inclusively masked by their minuends to ensure that only differences with respect to the activations of the first condition are evaluated.

In order to show areas that are shared by both processes, both these contrasts were entered into a conjunction analysis [S+>S− ∩ G+>G−], testing for independently significant effects compared at the same threshold (conjunction null, see [45]). This conjunction was inclusively masked by [S+ > baseline] and [G+ > baseline].

Areas activated to a stronger degree for the processing of gesture semantics as opposed to speech semantics were revealed by an interaction analysis [(G+>G−) > (S+>S−)], inclusively masked with (G+>G−) and (G+ > baseline). Correspondingly, areas activated to a stronger degree for the processing of speech semantics as opposed to gesture semantics were revealed by the following interaction contrast [(S+>S−) > (G+>G−)], inclusively masked with (S+>S−) and (S+ > baseline). The masking procedure was applied to ensure that differences between conditions are not a result of deactivation in a given contrast. Thus, all reported results reflect real activation increases with regard to the low level baseline (fixation cross).

We chose to employ Monte-Carlo simulation of the brain volume to establish an appropriate voxel contiguity threshold [46]. This correction has the advantage of higher sensitivity to smaller effect sizes, while still correcting for multiple comparisons across the whole brain volume. Assuming an individual voxel type I error of P<0.05, a cluster extent of 29 contiguous resampled voxels was indicated as necessary to correct for multiple voxel comparisons at P<0.05. This cluster threshold (based on the whole brain volume) has been applied to all contrasts and consequently is not affected by the masking procedure reported above. The reported voxel coordinates of activation peaks are located in MNI space. For the anatomical localization the functional data were referenced to probabilistic cytoarchitectonic maps [47].

## Results

### Behavioral Results

The average reaction time for the control task (“indicate the color of the spot on the actor's sweater”) did not differ across colors (F(1,15)  = 0.287, P<0.600) and conditions (F(3,45)  = 1.983, P<0.174, within-subjects ANOVA; m = 1.24 sec, SD  = 0.96). The participants showed an average accuracy rate of 99% which did not differ across conditions (F(3,45)  = 0.508, P = 0.619, within-subjects ANOVA). Thus, the attention control task indicates that participants did pay attention to the video clips.

### FMRI results

**Figure 2:**
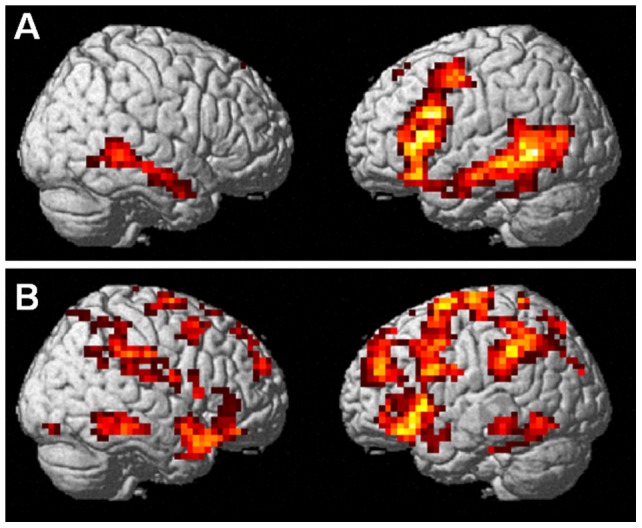
Within-modality semantic processing for speech (A; S+>S−) and iconic gestures (B; G+>G−).

Analyses targeting at within-modality semantic processing showed that language-related semantics as revealed by the contrast [S+>S−] were processed in a mainly left-lateralized network encompassing an extended frontotemporal cluster (inferior frontal gyrus, precentral gyrus, middle, inferior and superior temporal gyrus) as well as SMA in the left hemisphere and the right middle temporal gyrus (Table 1 and Figure 2a).

**Table 1:** Regions activated for familiar versus unfamiliar language [S+>S−] and for meaningful iconic versus control gestures [G+>G−].

Peak location		Cluster extension	BA	x	y	z	*t*-value	Extent
*Speech semantics [S+>S−]*								
L	MTG	STG, ITG, Hipp/Amyg	20, 21, 38	−64	−52	0	6.78	922
L	IFG	preCG, MFG	6, 9, 44, 45	−44	12	24	6.18	
R	MTG	ITG	21, 37	60	−40	−4	3.49	153
L	SFG	SMA	6	−8	20	48	2.98	37
*Gesture semantics [G+>G−]*								
L/R	Paracentral cortex	SMA, paracentral lobule, preCG, poCG, ACC, midCC, MFG	6, 3ab, 2, 4a	−4	−8	72	4.50	1.772
L	IFG	temporal pole, insula, Amyg	44, 45	−32	28	−8	4.22	
L	Parietal cortex	SPL, IPL, supra-marginal/angular gyrus, precuneus, MOG, SOG	7, 5	−56	−40	40	3.74	
R	Temporal Pole	insula, putamen, Hipp		36	8	−28	4.49	370
R	IFG	IFG, MOrbG	44, 45	48	36	−16	2.82	
L	Fusiform Gyrus	ITG, IOG, Hipp		−32	−28	−24	3.72	355
L/R	Basal ganglia	Thalamus, Pallidum, CN		−8	−24	0	3.41	
L	Rectal Gyrus			−8	16	−20	3.23	
R	ITG	ITG, PHG, Hipp, FusifG		28	−20	−24	3.98	144
R	Supramarginal gyrus	IPL, operculum, poCG	1, 3b, 40, 43	56	−36	40	3.34	139
R	MFG	prCG		40	12	56	3.25	41
R	SFG	SFG		20	60	28	3.12	34
R	FusifG	CalcG,	V3, BA 17/18	28	−80	−12	2.77	32

Note: Stereotactic coordinates in MNI space and *t*-values of the foci of maximum activation (P<0.05 corrected). Abbreviations: ACC = anterior cingulated cortex, Amyg = Amygdala, CalcG = calcarine gyrus, CN = caudate nucleus, FusifG =  fusiform gyrus, Hipp = Hippocampus, IFG = inferior frontal gyrus, IOG = inferior occipital gyrus, IPL = inferior parietal lobule, ITG = inferior temporal gyrus, MFG = Middle frontal gyrus, midCC = middle cingulated cortex, MOG = middle occipital gyrus, MOrbG = middle orbital gyrus, PHG = parahippocampal gyrus, preCG =  precentral gyrus, poCG = postcentral gyrus, SFG = superior frontal gyrus, SMA = supplementary motor area, SOG = superior occipital gyrus, SPL = superior parietal lobule STG = superior temporal gyrus.

Gesture-related semantics [G+>G−], in contrast, recruited a widely distributed bilateral network of regions (see Table 1 and Figure 2b).

### Common activations for semantics contained in iconic gestures and spoken language

**Figure 3:**
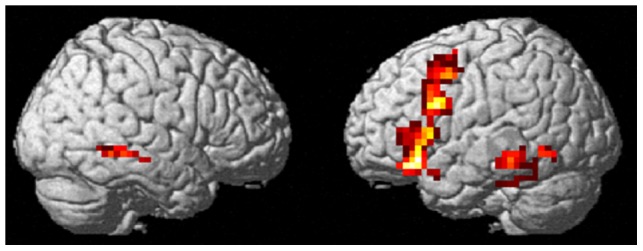
Common areas of activation for the processing of semantics derived from speech and iconic gestures (S+>S− ∩ G+>G−).

Semantic processing independent of input modality as disclosed by the conjunction of [S+>S− ∩ G+>G−] was related to a left-sided frontal cluster (extending from inferior frontal gyrus (BA 44, 45) across middle frontal gyrus and precentral gyrus), left inferior temporal cortex and right middle temporal gyrus (363 voxels; Table 2 and Figure 3).

**Table 2:** Regions activated for both speech and gesture semantics ([S+>S−] ∩ [G+>G−]).

Peak location		Cluster extension	BA	x	y	z	*t*-value	Extent
L	IFG	MFG, preCG, temporal pole	6, 44, 45	−40	28	−16	3.83	258
L	ITG	MTG/FusifG	20, 21,37	−60	−36	−16	3.06	75
R	MTG		20, 21,37	60	−36	−8	2.47	30

Note: Stereotactic coordinates in MNI space and *t*-values of the foci of maximum activation (P<0.05 corrected). Abbreviations: FusifG = fusiform gyrus, IFG = inferior frontal gyrus, ITG = inferior temporal gyrus, MFG = middle frontal gyrus, MTG = middle temporal gyrus, preCG = precentral gyrus.

### Interaction analyses: Activation differences between gesture and speech semantics

**Figure 4:**
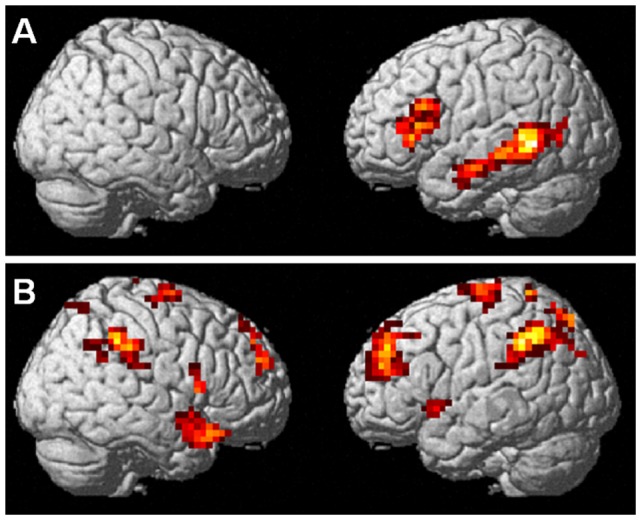
Stronger activations for speech semantics than gesture semantics (A; [S+>S−] > [G+>G−]) and vice versa (B; [G+>G−] > [S+>S−]).

Speech semantics elicited significantly stronger activations than gesture semantics [(S+>S−) > (G+>G−)] along the left middle temporal gyrus and in the left inferior frontal gyrus (pars triangularis, BA 44, 45; 291 voxels; Table 3 and Figure 4A).

**Table 3:** Regions activated specifically for speech and gesture semantics.

Peak location		Cluster extension		BA	x	y	z	*t*-value	Extent
*Speech semantic > gesture semantic ([S+>S−] > [G+>G−])*									
L	MTG	ITG, MOG	21,20, 19,37		−60	−52	0	4.85	195
L	IFG	IFG	45		−48	28	4	3.32	96
*Gesture semantic > Speech semantic ([G+>G−]> [S+>S−])*									
L/R	SPL	Precuneus, SMA, midCC, SOG, preCG, poCG, IPL	2, 3, 5, 6, 7		−12	−64	60	4.21	549
R	Insula	STG, IFG, Hipp, Putamen, Pall	44, 38,47		36	12	−24	4.47	260
L	IPL	Angular-/supramarginal gyrus	7, 40		−48	−44	40	4.37	133
L	SFG	MFG	9, 10, 46		−24	52	40	5.56	113
L	Parahippo-campal gyrus	Thalamus			−24	−40	−8	3.92	110
R	Supramarginal gyrus		40		56	−40	44	5.52	80
R/L	Medial cluster	SIG, ACC	8, 32		4	44	44	3.32	63
L	N.A.	olfactoric cortex/Hypothalamus			−8	4	−12	3.75	40
R	SFG		10		24	48	20	5.33	33
L	Insula				−36	8	4	3.10	30

Note: Stereotactic coordinates in MNI space and *t*-values of the foci of maximum activation (P<0.05 corrected). Abbreviations: IFG = inferior frontal gyrus, IPL = inferior parietal lobule, ITG = inferior temporal gyrus, midCC = middle cingulated cortex, MOG = middle occipital gyrus, MTG = middle temporal gyrus, preCG =  precentral gyrus, poCG = postcentral gyrus, SMA = supplementary motor area, SOG = superior occipital gyrus, SPL = superior parietal lobule, STG = superior temporal gyrus, N.A. = Not assigned.

The inverted contrast [(G+>G−) > (S+>S−)] revealed that gesture semantics activated a more widespread bilateral network, comprising superior and inferior parietal regions, superior frontal and medial areas, right temporal pole and left insula (Table 3 and Figure 4B than speech semantics).

## Discussion

We hypothesized that the semantic processing of spoken language and iconic gestures is based on a common neural network. Our study design tailored the comparison to the level of semantics, controlling for lower processing levels such as sound and motion kinematics. Thus, this study was basically focused on the feature extraction level of the feature integration model [35]. The results demonstrate that the pathways engaged in the processing of semantics contained in both spoken language and iconic gestures comprise the left IFG, the left inferior temporal and the right middle temporal gyrus. Thus, in line with our hypothesis we found modality-independent activation in a bilateral frontotemporal network with a leftwards asymmetry.

### Processing of speech semantics

The results of the speech contrast [S+>S−] are in line with other studies that contrasted the processing of a native against an unknown foreign language [48]–[50]. We found activation along the left temporal lobe (including STG, MTG and ITG), in the LIFG extending into the precentral gyrus, and along the right MTG. This strongly left-lateralized pattern has been found in all of the above mentioned studies. Apart from these studies with conditions very similar to our study, temporal as well as inferior frontal regions have been frequently implicated in various kinds of language tasks (for reviews see [1], [2], [51]). For the LIFG Hagoort (2005) identified an anterior-ventral to posterior-dorsal gradient, with BA 47 and BA 45 contributing to semantic processing, BA 45 and BA 44 processing syntactics and BA 44 and parts of BA 6 playing a role in phonological processing [52] – all of these regions have been revealed by our [S+>S−] contrast. Most likely this contrast uncovers not only activation related to semantic, but also to syntactic (as the syntax of Russian speech could not be evaluated by our subjects) and phonological processing (as the speech sounds of Russian language are different from those of German language). The temporal regions found in our analysis have been related to the storage and retrieval of linguistic information, specifically for semantic information (see e.g., [53]–[55]). These temporal semantic regions have been shown to consist of category-specific and spatially separable subdivisions, with regions relating to persons, animals or tools (for a review see [1]).

These fronto-temporal areas classically associated with language processing seem to be stronger activated by speech semantics than by semantics evoked by gestures as indicated by our interaction analysis. Within this analysis we found that processing of speech semantics in contrast to gesture semantics relied on frontal (LIFG) and temporal regions (MTG). Thus, despite the finding of a supra-modal network including especially left inferior frontal and posterior temporal regions (see below), parts of these regions are more involved in the processing of speech semantics in contrast to gesture semantics.

### Processing of gesture semantics

In line with studies on action observation (e.g., [22], [56]–[58]) we found for the processing of gesture semantics a bilaterally distributed network of activation including the premotor cortex, inferior and middle frontal gyri, inferior temporal gyrus and parietal regions.

However, the semantic aspects of the iconic gestures used in the present study differed from all previous studies with regard to the type of information and the specificity of the presented content: Previous studies have either presented pantomimes of tool or object use, hands grasping for tools or objects (e.g., [22], [56]–[65]) or have shown symbolic gestures like “thumbs up” [21], [66]–[68]. Our stimuli in contrast consisted of iconic gestures that normally are used to accompany speech (e.g., [19], [25], [69]–[72]). Thus, compared to symbolic gestures, iconic gestures are less clear in their meaning when presented without speech. Despite these differences in stimuli we found a similar network of activations as in a previous study [21], suggesting that even ambiguous gesture information activates semantic representations. The remarkable distributed activation pattern in our study most likely is due to this more diffuse meaning, reflecting enhanced decoding processing effort to enable understanding. However, our findings provide a first support for the assumption that some aspects of semantic information are extracted from iconic gestures already at the feature extraction level [35].

Interaction contrasts revealed that processing of the less apparent gesture meaning compared to speech semantics engaged a broader network that included parietal regions, superior frontal regions and sensorimotor areas. All of these areas have previously been related to action processing (e.g., [56], [59], [60], [63], [73]–[77]) and seem to process semantics derived from gestures.

### Supramodal semantic processing

The processing of spoken language semantics and semantic information conveyed through iconic gestures activated an overlapping network of brain regions including the left inferior frontal cortex (BA 44, 45) expanding into the precentral gyrus (BA 4, 6), the left inferior temporal gyrus (BA 37) and a smaller cluster in the right middle temporal gyrus, suggesting the existence of a supramodal semantic network.

These results extend studies from both the gesture and the language domain (see above) in showing a common neural representation of speech and iconic gesture semantics. Furthermore, the findings go beyond previous reports about common activation between symbolic gestures and speech semantics [21], in showing comparable effects for less conventionalized and less language like iconic gestures. However, differences in the sub-regions of the left IFG and posterior temporal lobe between the present findings and results for symbolic gestures [21], suggest an specific involvement of the motor-cortex and the more inferior part of the temporal lobe in the processing of iconic gesture and speech semantics. These results suggest a high flexibility of the supramodal network, recruiting specific subregions of the left IFG and posterior temporal lobes dependent on content and specificity of communicated meaning. The left-lateralization of our findings is congruent with the majority of fMRI studies on language (see [1], [51], for reviews). But the right hemisphere makes substantial contributions to communication such as keeping track of the topic or drawing inferences from utterances [3], [78]. Left fronto-temporal activations have been frequently observed for semantic processing (e.g., [79]; for a review see [2]), for the decoding of meaningful actions (e.g., [22], [58]) and also with regard to co-verbal gesture processing [26], [28]–[32].

With regard to the inferior frontal activations, functional imaging studies have underlined the importance of this region in the processing of language semantics. The junction of the precentral gyrus and the pars opercularis of the LIFG has been involved in controlled semantic retrieval [80]–[82], semantic priming [83]–[88] and a supramodal network for semantic processing of words and pictures [83]. The middle frontal gyrus (MFG) was found activated by intramodal semantic priming (e.g., [89]) and the right inferior frontal gyrus demonstrated response suppression in crossmodal semantic priming [83]. In addition, knowledge relating to manipulable objects has repeatedly been located in the precentral gyrus (for reviews, see [90], [91]). Studies on gesture processing constantly have found Broca's area/LIFG/ventral premotor cortex stronger activated for meaningful (e.g. transitive pantomimes) compared to meaningless gestures (see meta-analysis by [58]). Fadiga and colleagues (2006) have demonstrated that the activation of the classic motor speech centre in action observation is genuine and not due to verbalization processes [92]. The activations that we observed in the inferior and middle temporal gyrus most likely reflect the retrieval of conceptual information derived from both information channels. A meta-analysis of 120 functional imaging studies by Binder and colleagues (2009) recapitulated that the posterior temporal cortex constitutes a multimodal and heteromodal association cortex. Especially the posterior proportions have been found activated irrespective of whether the stimuli (e.g., objects) were presented as pictures, written or spoken language [93]. We found a more inferior region of the temporal lobe and not the angular gyrus to be activated by speech and gesture semantics. The angular gyrus, however, seems to be more involved in the processing of gesture semantics [G+>G− > S+>S−]. Thus, our results suggest that stimulus triggered semantic processes that are common to the speech and gesture domain might rather rely on inferior frontal and inferior/middle temporal brain regions. In line with the mentioned meta-analysis, these regions seem also to have supramodal properties (i.e., are activated by semantic tasks across visual and auditory modalities [93]).

Since semantic memory is the basis of semantic processing, an amodal semantic memory [94] is a likely explanation for how speech and gesture could activate a common neural network. Our findings suggest supramodal semantic processing in regions including the left temporal pole, which has been described as best candidate for a supramodal semantic “hub” [94]. Thus, semantic information contained in speech and gestures might have activated supramodal semantic knowledge in our study. Importantly, despite subjects performed a non-semantic control task (pressing one of two buttons, depending on the color of the spot on the sweater), we found that speech and gesture semantics (meaningful > meaningless) were processed in overlapping neural structures. This indicates that features of speech and gesture are able to trigger semantic processing/knowledge (bottom up). An alternative explanation for our findings could stem from differences in familiarity between conditions. However, contrarily to this assumption is the fact that familiarity usually leads to reduced neural responses in contrast to novel/unfamiliar or mismatching information (e.g. for action observation [95], speech or co-verbal gesture processing [25], [29]). Thus, an opposite pattern of activation (meaningless/unfamiliar > meaningful/familiar) would be expected based on differences in familiarity.

Our results are extending the findings of Xu and colleagues (2009) who examined symbolic gestures and their spoken analogies and identified the left posterior MTG and superior temporal sulcus, the left IFG and the right posterior MTG as areas of common activation [21]. The high consistence in results between our and their study is remarkable, considering the different kinds of stimuli used: Whereas Xu and co-workers used highly conventionalized gestures and pantomimes often including prompts to the viewer (“settle down!”; “thumbs up”), we used non-conventionalized iconic gestures that are used only in combination with speech and describe properties of actions or objects. Symbolic stimuli like the emblems used in the Xu study bear no formal relationship with the content of the utterance they accompany; their meaning is clear-cut and highly overlearned. Thus, it is not surprising that such a learned meaning is represented in a neural network overlapping with the corresponding language representations.

In our study the gestures' meaning was less specific and novel to the participants, but still activated brain regions overlapping with the processing of speech semantics. Thus, our findings provide a first support for the assumption that at least some aspects of semantic information are extracted from iconic gestures already at the feature extraction level [35]. Additional resources required for a more intensive search for meaning might explain the activation of the precentral gyrus present in our, but not in the Xu study. Thus, activation of the motor cortex might be relevant for extracting meaning of complex movements with unspecific meaning. It might as well be possible that isolated hand gestures without a clear meaning are initially interpreted as relating to any kind of object manipulation, as this is what our hands are made for. It is well known that object knowledge also includes associations with sensorimotor correlates of their use, i.e. motor programs stored in pericentral regions. This explanation would be indicative of a common origin of motor behavior and semantic knowledge. Concerning the speech stimuli there was another difference between the two studies: While Xu et al. used words and digitally modified pseudowords we presented short sentences and their translations into Russian, i.e. we used more complex and, importantly, in the control condition more natural stimuli. Thus, differences in activation pattern between studies might be due to these differences in control conditions.

In addition to the differences in frontal activation our results also suggest a more inferior part of the posterior temporal lobe (compared to results of Xu et al.) to be involved in the common semantic network identified for iconic gestures and concrete speech. Left inferior and middle temporal activations have been reported for meaningful speech comprehension [51] and semantic retrieval [93]. Furthermore, the inferior temporal gyrus has been found for amodal semantic processing [96] and the maintenance of conceptual information [8]. Thus, depending on gesture type (iconic vs. emblematic) different aspects of supramodal semantic processes seem to be involved in extracting meaning from speech and gesture. Future studies are necessary to disentangle the function of inferior and superior aspects of the posterior temporal lobe in the processing of semantic information contained in emblematic and iconic gestures.

### Implications

In the past, all of the revealed areas have been related to the network associated with different aspects of language comprehension. All of them have been shown part of a network contributing to semantic processing of written, spoken and signed language, for example by lexical storage and access (MTG, ITG), retrieval and selection of lexical information (IFG) (see [97], for review). The interplay of these regions enables the integration of different representations into a continuously developing semantic context – independent of modality. Our results support the hypothesis that these former findings are not limited to language, be it written, spoken or signed. We could demonstrate activation of a supramodal network for speech semantics and unspecific and hard to verbalize iconic gestures semantics. The identified fronto-temporal network maps not only sound and meaning in the auditory domain but also combines gestures and their meanings in the gestural-visual domain. This modality-independent network most likely gets input from modality-specific areas in the superior (speech) and inferior temporal lobe (gestures) where the main characteristics of the spoken and gestured signals are decoded. The inferior frontal regions are responsible for the process of selection and integration, relying on more general world knowledge distributed throughout the brain [21].

This is somewhat contradictory to studies on speech gesture integration where the left IFG has not been found consistently [25], [28], [31], [32]. These studies rather suggest that the role of the IFG in speech gesture integration processes is not purely integrative but rather related to the detection and resolution of incompatible stimulus representations (as in mismatch designs like [29]) and for implementing reanalyses in the face of misinterpretations [98], [99]. This explanation might also account for IFG involvement in the processing of metaphoric speech-gesture pairs where the speech content cannot be taken literally (if it was taken literally there would be conflict between speech and gesture) and has to be transferred to an abstract level [26]–[28]. Instead, a region at the temporo-occipital junction seems to fulfill the integration of speech and iconic gestures in a natural context [25], [27], [28], [31]. Taken together, for speech gesture processing our results rather assigns the LIFG a semantic-related processing step just before integration.

Our findings also corroborate the theory about the evolutionary origins of human communication [21], [100]–[102]: It is assumed that a precursor of the here presented fronto-temporal system supported gestural communication by pairing gesture and meaning. As voluntary control over the vocal apparatus evolved and spoken language developed this system was then adapted for the comparable pairing of sound and meaning, keeping its original function in gesture processing [21].

### Conclusion

In the last years the understanding of speech and gesture processing has increased, both communication channels have been disentangled and again were brought together. But so far there had been a “missing link” in the research along the continuum between symbolic gestures, speech-accompanying gestures like iconic gestures and isolated speech. Our study bridges this gap and provides evidence that there is a common and thus amodal neural system for the processing of semantics contained in language and gestures. The challenge for future studies will be the identification of specific aspects of speech and gesture semantics or the respective format relevant for the understanding of the role of specific sub-regions of the left IFG and the posterior temporal lobes.
